# Initial clinical experience with [^177^Lu]Lu-PNT2002 radioligand therapy in metastatic castration-resistant prostate cancer: dosimetry, safety, and efficacy from the lead-in cohort of the SPLASH trial

**DOI:** 10.3389/fonc.2024.1483953

**Published:** 2025-01-07

**Authors:** Aaron R. Hansen, Stephan Probst, Jean-Mathieu Beauregard, Benjamin L. Viglianti, Jeff M. Michalski, Scott T. Tagawa, Oliver Sartor, Ronald F. Tutrone, Orhan K. Oz, Kevin D. Courtney, Ebrahim S. Delpassand, Luke T. Nordquist, Medhat M. Osman, Kim N. Chi, Richard Sparks, Noble George, Sara M. Hawley, Wenting Wu, Jessica D. Jensen, Neil E. Fleshner

**Affiliations:** ^1^ Department of Medical Oncology, Princess Margaret Cancer Centre, Toronto, ON, Canada; ^2^ Department of Nuclear Medicine, Jewish General Hospital, Montreal, QC, Canada; ^3^ Department of Medical Imaging, Center Hospitalier Universitaire (CHU) de Québec – Université Laval, Quebec City, QC, Canada; ^4^ Department of Radiology, Nuclear Medicine Division, University of Michigan, Ann Arbor, MI, United States; ^5^ Department of Radiation Oncology, Washington University School of Medicine, Saint Louis, MO, United States; ^6^ Department of Medicine, Weill Cornell Medical Center, New York, NY, United States; ^7^ Department of Oncology, Mayo Clinic Rochester, Rochester, MN, United States; ^8^ Chesapeake Urology Associates (CUA) P.A., Towson, MD, United States; ^9^ Department of Radiology, University of Texas (UT) Southwestern, Dallas, TX, United States; ^10^ Department of Internal Medicine, University of Texas (UT) Southwestern, Dallas, TX, United States; ^11^ Excel Diagnostics and Nuclear Oncology Center, Houston, TX, United States; ^12^ Urology Cancer Center, PC, Omaha, NE, United States; ^13^ Department of Radiology, Division of Nuclear Medicine, Saint Louis University Hospital and St. Louis VA Medical Center, St. Louis, MO, United States; ^14^ Department of Medicine, University of British Columbia, Vancouver, BC, Canada; ^15^ CDE Dosimetry Services, Knoxville, TN, United States; ^16^ Department of Clinical Development, POINT Biopharma, a wholly owned subsidiary of Eli Lilly and Company, Indianapolis, IN, United States

**Keywords:** castration resistant prostate cancer, PSMA, radioligand therapy, PNT2002, dosimetry

## Abstract

**Introduction:**

SPLASH (NCT04647526) is a multicenter phase III trial evaluating the efficacy and safety of [^177^Lu]Lu-PNT2002 radioligand therapy in metastatic castration-resistant prostate cancer (mCRPC). This study leveraged a lead-in phase to assess tissue dosimetry and evaluate preliminary safety and efficacy, prior to expansion into a randomized phase. Here we report those results.

**Methods:**

Enrolled participants had mCRPC that progressed on one prior androgen receptor pathway inhibitor (ARPI), were prostate-specific membrane antigen (PSMA) PET–positive as determined by a central reader, were chemotherapy-naïve for mCRPC, and had adequate bone marrow and end-organ reserve. Participants received up to 4 cycles of [^177^Lu]Lu-PNT2002 at 6.8 GBq (± 10%) intravenously per cycle every 8 weeks. Dosimetry (planar + SPECT/CT [n=7]; planar only [n=20]), safety, prostate-specific antigen (PSA) response, objective response rate (ORR), and radiographic progression-free survival (rPFS) per blinded independent central review were assessed.

**Results:**

Of 34 individuals screened, 32 underwent PSMA-PET/CT; 27 met all eligibility criteria. Median (range) age was 72 (57-86) years; all participants were enrolled in North America; 40.7% initiated prior ARPI treatment without distant metastases (M0) and 25.9% while hormone sensitive. Nineteen of 27 (70.4%) participants completed all 4 planned cycles. Organs receiving the largest mean (median, range) specific absorbed doses were lacrimal glands at 1.2 (0.9, 0.4-6.7) Gy/GBq (planar only [n=27]), followed by kidneys at 0.73 (0.63, 0.22-1.8) Gy/GBq (planar + SPECT/CT [n=7]; planar only [n=20]). Mean (median, range) tumor specific absorbed dose was 4.3 (2.1, 0.3-33.4) Gy/GBq (approximately 29 Gy/cycle) based on planar + SPECT/CT of 21 lesions in seven participants. [^177^Lu]Lu-PNT2002 was associated with no treatment-related deaths, few treatment-related grade ≥3 treatment-emergent adverse events (TEAEs), and no discontinuations for unacceptable toxicity. Treatment-related TEAEs occurring in ≥10% of participants included dry mouth (22.2%; all grade 1), fatigue (18.5%; grades 1-2), nausea (18.5%; grades 1-2), and anemia (14.8%; grades 1-3). Median (95% CI) rPFS was 11.5 (9.2-19.1) months, a PSA decline of ≥50% occurred in 42.3% (11/26) of participants, and confirmed ORR for evaluable disease was 50% (5/10).

**Conclusion:**

[^177^Lu]Lu-PNT2002, administered at 6.8 GBq/cycle for 4 cycles, demonstrated a favorable dosimetry and safety profile, as well as promising preliminary efficacy.

**Clinical trial registration:**

https://clinicaltrials.gov/, identifier NCT04647526.

## 1 Introduction

Despite recent treatment advances, prostate cancer remains a leading cause of cancer death among males globally (1). The vast majority of deaths occur in patients with advanced metastatic castration-resistant prostate cancer (mCRPC). A common 1st line treatment for mCRPC is to add an androgen receptor pathway inhibitor (ARPI; e.g., abiraterone, apalutamide, darolutamide, or enzalutamide) to ongoing androgen deprivation therapy (achieved through surgical or chemical castration) (2). Approved treatment options following initial ARPI failure include chemotherapy, sipuleucel-T, radium-223, and poly ADP ribose polymerase inhibitors (3–7). Unfortunately, many of these options are only indicated for subsets of individuals (5–7) or may have a suboptimal toxicity-to-efficacy ratio for many with mCRPC (8–10). A common treatment pathway is thus a second line ARPI, despite limited clinical benefit and short duration of response (10). Although a prostate-specific membrane antigen (PSMA)-targeted radioligand therapy was approved by the Food and Drug Administration for mCRPC in 2022, it is only indicated for patients previously treated with chemotherapy, thereby excluding a large proportion of patients who are unfit or unwilling to receive chemotherapy (9). Hence, there is a substantial unmet medical need for therapies with demonstrated effectiveness and favorable safety profiles.

[^177^Lu]Lu-PNT2002 consists of a beta-emitting [^177^Lu]lutetium radioisotope chelated to a small-molecule ligand (also known as PSMA-Imaging & Therapy [I&T]) that targets PSMA using a glutamate urea-based pharmacophore, enabling targeted delivery of radiation to prostate cancer cells, causing DNA damage and ultimately cancer cell death. [^177^Lu]Lu-PSMA-I&T used as an investigational agent has demonstrated promising anti-tumor activity with limited side effects (11).

SPLASH (**S**tudy Evaluating Metastatic Castrate Resistant **P**rostate Cancer Treatment Using [^177^Lu]**L**u-PNT2002 PSMA Therapy **A**fter **S**econd-line **H**ormonal Treatment; NCT04647526) is a prospective, multicenter, phase III, open-label, randomized trial evaluating the efficacy and safety of [^177^Lu]Lu-PNT2002 in patients with mCRPC. The trial commenced with a safety and dosimetry lead-in portion, prior to proceeding into the larger randomized portion, which enrolled over 400 participants across 55 sites. Herein, we report the results of the lead-in portion, which evaluated dosimetry, safety, and efficacy to confirm that the planned administered activity elicited the anticipated anti-tumor response without exhibiting unexpected clinical toxicity or surpassing pre-specified absorbed dose tolerances to critical organs.

## 2 Materials and methods

### 2.1 Study design

This prospective study was approved by the institutional review board for each participating site and conducted in accordance with Good Clinical Practice and the Declaration of Helsinki. All participants provided written informed consent.

### 2.2 Study population

Participants were enrolled across 12 sites, had been diagnosed with progressive mCRPC (by prostate-specific antigen [PSA], bone scan and CT/MRI, as per Prostate Cancer Working Group 3 [PCWG3] criteria (12), had experienced disease progression on a single prior ARPI, had adequate bone marrow and end organ reserve, and were PSMA PET/CT scan ([^68^Ga]Ga-PSMA-11 or [^18^F]F-DCFPyL)–positive as determined by a central reader. For measurable disease (per Response Evaluation Criteria in Solid Tumors [RECIST] v1.1), threshold standard uptake value (SUV)_max_ had to be ≥15 at ≥1 site and >10 at all measurable sites; in the absence of measurable disease, one site with an SUV_max_ >10 was required. Patients with central nervous system metastases, liver metastases >1 cm, a superscan on bone scintigraphy, or prior cytotoxic chemotherapy for mCRPC were excluded. Prior chemotherapy for hormone-sensitive prostate cancer was permitted if the last dose was administered >1 year prior to consent.

### 2.3 Dosing and endpoints evaluation

Participants were treated with [^177^Lu]Lu-PNT2002 at 6.8 GBq (± 10%) intravenously (IV) every 8 weeks for up to 4 cycles. Health authority guidance was provided to optimize the safety profile by minimizing renal injury to a 5% risk threshold modeled from EBRT estimates (13). Key endpoints included dosimetry, safety, radiographic progression-free survival (rPFS), overall survival (OS), radiographic objective response rate (ORR), and PSA response. PSA was assessed every 4 weeks. CT/MRI and whole-body bone scintigraphy were conducted every 8 weeks until progression, with rPFS and ORR assessed by blinded independent central review per RECIST v1.1 for soft tissue and PCWG3 criteria for bone. Radiographic PFS was defined as time from enrollment to progression by blinded independent central review or death from any cause; participant data were censored in cases of withdrawal of consent or initiation of an alternative anticancer treatment prior to centrally confirmed radiographic progression. Confirmed ORR was defined as the number of participants with confirmed complete or partial responses divided by the number with evaluable disease (defined as RECIST v1.1 target and/or non-target lesions) at baseline. Confirmed PSA response rate was defined as the proportion of participants achieving a ≥50% decrease in PSA from baseline to the lowest post-baseline PSA result (PSA_50_), confirmed by a second PSA reduction ≥50% at least 3 weeks later. Adverse events were reported using Common Terminology Criteria for Adverse Events v5.0. Treatment-related adverse events include events assessed by the investigator as definitely, probably, or possibly related to treatment. Long-term follow-up is at least five years from first therapeutic administration, or until participant is lost to follow-up or dies.

### 2.4 Image acquisition and dosimetry

For normal organ dosimetry, whole-body planar images were collected for all participants at five time points following the first treatment: 0.5-2 h (pre-void), 24 h (± 4 h), 48 h (± 4 h), 72 h (± 4 h), and 140-196 h. Region of interest (ROI) construction was manual and included a heart ROI to determine blood activity for red marrow estimation, scatter correction was done using a triple energy window technique, data were decay corrected and fit in a least squares sense using non-linear regression with sums of exponentials, and OLINDA/EXM v2.2 software was used to estimate absorbed doses (see **Supplementary Materials** for expanded methods). Estimated cumulative absorbed doses after four cycles were extrapolated from cycle 1 dosimetry results.

In addition to the whole-body planar imaging, abdominal SPECT/CT was obtained at 48 ± 4 h post injection. SPECT/CT images that met prespecified quality control standards were utilized to determine kidney, red marrow (based on a lumbar spine ROI), and tumor activity at the SPECT/CT image time and results were used to scale the planar biodistribution curves for the corresponding participants according to the standard hybrid (planar + SPECT/CT) method (see **Supplementary Materials** for expanded methods).

### 2.5 Statistical analysis

Target enrollment for the lead-in portion of SPLASH was 25 participants; statistical analyses were descriptive in nature. The survival follow-up, defined as the duration between the enrollment date and the last known alive date/death date (in months), was analyzed using the reverse Kaplan-Meier method (i.e., with censoring at the date of death for those who died). The median follow-up time and its corresponding 95% CI are summarized. OS, defined as the time from the enrollment date to the date of death from any cause, was analyzed using the Kaplan-Meier method. Subjects who did not die at the time of the analysis were censored at the date the subject was last known to be alive.

## 3 Results

**Figure 1:**
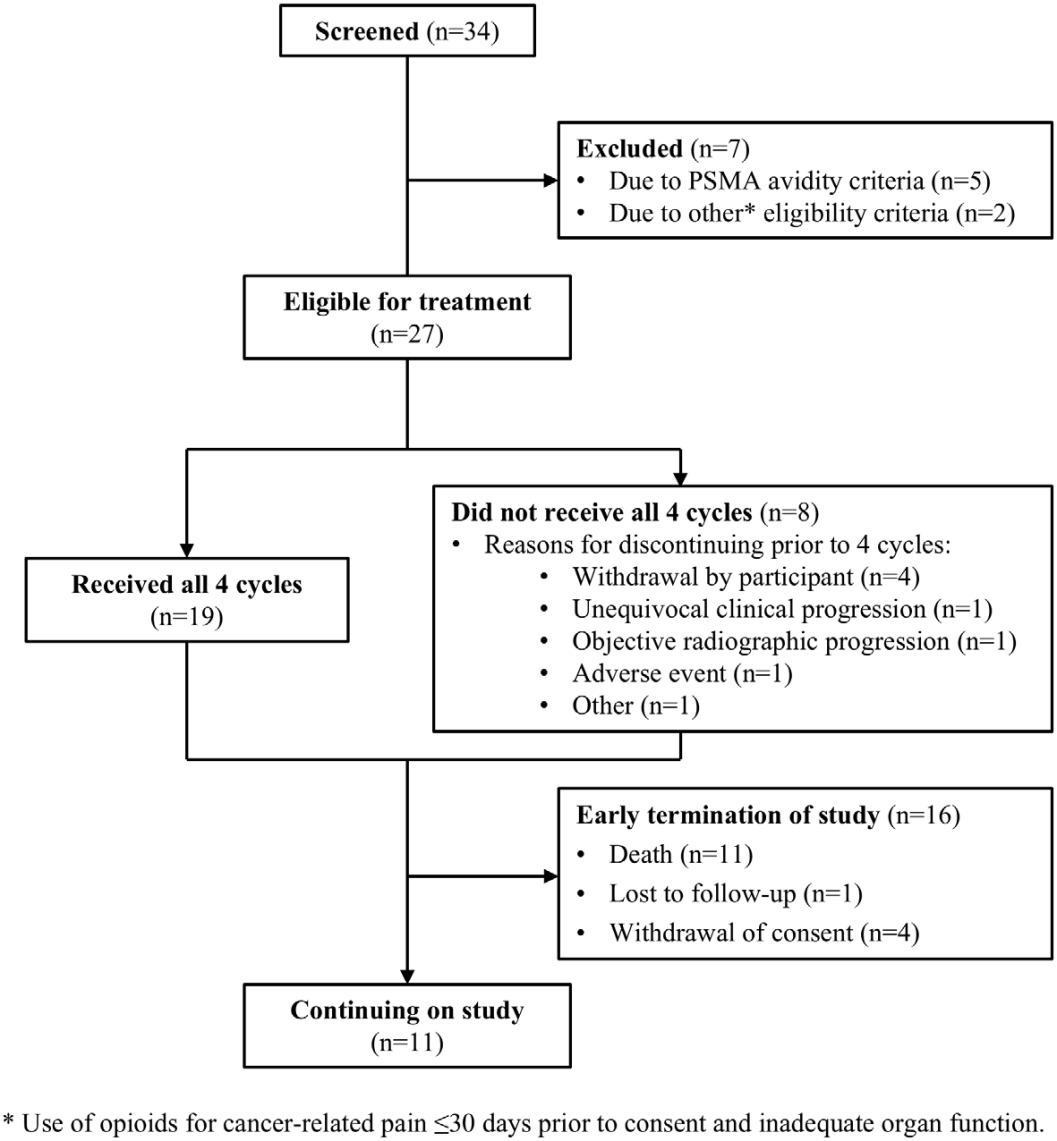
Participant disposition for the SPLASH trial lead-in cohort.

Of 34 individuals screened, 32 underwent baseline PSMA-PET/CT. Of those, 84.4% (27/32) were PSMA-positive and met all study eligibility criteria (**Figure 1**). Participants had a median (range) age of 72 (57-86) years, 85.2% (23/27) were White, 14.8% (4/27) were Black or African American, and 22.2% (6/27) had previously received chemotherapy for hormone-sensitive prostate cancer (**Table 1**). At the time of initial APRI initiation, 25.9% (7/27) were hormone-sensitive and 40.7% (11/27) did not have distant metastases by conventional imaging.

**Table 1:** Baseline participant demographics and characteristics.

	Participants (n=27)
Age at informed consent, years	
Mean (SD)	71.4 (8.5)
Median (range)	72.0 (57 – 86)
Age group, n (%)	
<65 years	7 (25.9)
≥65 years	20 (74.1)
Race, n (%)	
Black or African American	4 (14.8)
White	23 (85.2)
Country, n (%)	
Canada	12 (44.4)
United States	15 (55.6)
PSA (μg/L) at baseline, median (range)	22 (0.3 – 701.0)
ECOG performance status, n (%)	
0	16 (59.3)
1	11 (40.7)
Sites of metastases, n (%)	
Node	11 (40.7)
Bone	24 (88.9)
Other (bladder, seminal vesicles, and rectum)	1 (3.7)
Prior taxane treatment for hormone-sensitive prostate cancer, n (%)	
Yes	6 (22.2)
No	21 (71.8)
Prior bone health treatment*, n (%)	
Yes	14 (51.9)
No	13 (48.1)
Receiving opioids for cancer-related pain, n (%)	
No	27 (100.0)
Prior ARPI therapy, n (%)	
Yes	27 (100.0)
Abiraterone	12 (44.4)
Enzalutamide	12 (44.4)
Apalutamide	2 (7.4)
Darolutamide	1 (3.7)
Prior ARPI indication, n (%)	
Non-metastatic CRPC	11 (40.7)
Metastatic hormone-sensitive prostate cancer	7 (25.9)
Metastatic CRPC	9 (33.3)

*Denosumab or zoledronic acid.

Participants received a median of 4 cycles of [^177^Lu]Lu-PNT2002 with 70.4% (19/27) completing all 4 planned cycles (**Table 2**). Reasons for treatment discontinuation included disease progression (n=2), fatal event of disseminated intravascular coagulation assessed by investigator and sponsor as not related to treatment (n=1), sponsor request (n=1; previously undiagnosed condition precluded study eligibility), and withdrawal by participant (n=4; reasons included initiated alternative treatment, transferred to a different hospital, and moved to a different state; in one case, no reason was provided). The median administered activity per cycle was 6.9 (6.2-7.5) GBq.

**Table 2:** Treatment exposure.

	Participants (n=27)
Number (%) of participants by number of [^177^Lu]Lu-PNT2002 cycles	
1 cycle	3 (11.1)
2 cycles	3 (11.1)
3 cycles	2 (7.4)
4 cycles	19 (70.4)
Activity per cycle (GBq) of [^177^Lu]Lu-PNT2002	
Total number of cycles	91
Mean (SD)	6.9 (0.3)
Median (range)	6.9 (6.2 – 7.5)

### 3.1 Dosimetry

Radiation dosimetry was calculated in all 27 participants based on biodistribution data from planar whole-body conjugate imaging. Additionally, quantitative SPECT/CT images that met prespecified quality control standards were available for seven participants. These were used for renal, red marrow, and tumor dosimetry.

SPECT/CT and planar-based kidney activity estimates (at the time of the SPECT/CT) were consistent (± 20%) across six of the seven participants where SPECT/CT was available (**Supplementary Table 1**). For the remaining participant where SPECT/CT was acquired, the kidneys were completely obscured by overlap of gastrointestinal activity in the planar images. Hybrid (planar + SPECT/CT) kidney dosimetry results (n=7) were combined with the planar-only kidney results (n=20) to calculate summary statistics for the kidney dose.

Organs receiving the largest mean (median, range) specific absorbed doses were the lacrimal glands at 1.2 (0.9, 0.4-6.7) Gy/GBq, followed by the kidneys at 0.73 (0.63, 0.22-1.8) Gy/GBq (**Table 3**). The mean (median, range) dose to the salivary glands was 0.34 (0.25, 0.14-1.5) Gy/GBq. The mean (median, range) dose to red marrow was 0.033 (0.037, 0.010-0.053) Gy/GBq using the planar-SPECT/CT hybrid imaging methodology (n=7) and 0.034 (0.027, 0.014-0.11) Gy/GBq using the heart ROI, blood-based methodology (n=27). Tumor lesions received a mean (median, range) dose of 4.3 (2.1, 0.3-33.4) Gy/GBq (n=21 lesions from 7 participants). For the 27.2 GBq cumulative administered activity (4 cycles x 6.8 GBq/cycle), the estimated mean cumulative absorbed dose extrapolated from cycle 1 was 117 Gy for tumor lesions, 19.9 Gy for kidneys, and 0.9 Gy for red marrow. At the individual level, the extrapolated cumulative renal dose exceeded 23 Gy in 22.2% (6/27) of participants.

**Table 3:** [^177^Lu]Lu-PNT2002 normal organ and tumor lesion dosimetry.

		Specific Absorbed Dose Estimate		4-Cycle* Cumulative Absorbed Dose Estimated Mean (Gy)
Organ	Imaging Type (n)	Mean ± SD (Gy/GBq)	Median, Range (Gy/GBq)	
Kidneys	Planar (20[19^†^]) and hybrid^‡^ (7)	0.73 ± 0.33 0.69 ± 0.25^†^	0.63, 0.22-1.8 0.63, 0.22-1.5^†^	19.9 18.8
Lacrimal glands	Planar (27)	1.2 ± 1.2	0.94, 0.38-6.7	32.6
Left colon	Planar (27)	0.52 ± 0.28	0.47, 0.013-1.5	14.1
Liver	Planar (27)	0.051 ± 0.042	0.041, 0.025-0.24	1.4
Red marrow				
Lumbar spine-based Heart/blood-based^§^	Hybrid (7) Planar (27)	0.033 ± 0.014 0.034 ± 0.019	0.037, 0.010-0.053 0.027, 0.014-0.11	0.9 0.9
Salivary glands	Planar (27)	0.34 ± 0.27	0.25, 0.14-1.5	9.2
Spleen	Planar (27)	0.16 ± 0.17	0.10, 0.022-0.80	4.4
Thyroid	Planar (27)	0.17 ± 0.15	0.13, 0.011-0.77	4.6
Tumor lesions (n=21 lesions)	Hybrid (21)	4.3 ± 7.0	2.1, 0.32-33.4	117.0

*6.8 GBq/cycle. ^†^Excluding participant with hypoplastic right kidney and undiagnosed acute renal failure prior to first cycle due to disease obstruction of left ureter. ^‡^Planar + SPECT/CT. ^§^Method described in **Supplementary Materials.**

### 3.2 Safety

[^177^Lu]Lu-PNT2002 was well tolerated with no treatment-related treatment-emergent adverse events (TEAEs) leading to death or discontinuation (**Table 4**). The majority of TEAEs were grade 1 or 2 (**Table 5**). Collectively, myelosuppression TEAEs occurred in 29.6% (8/27) of participants and were considered treatment-related in 18.5% (5/27). The most common treatment-related TEAEs were dry mouth (22.2%; 6/27; all grade 1), fatigue (18.5%; 5/27), nausea (18.5%; 5/27), and anemia (14.8%; 4/27). There was a single grade 4 event (hyponatremia) and a single fatal event (disseminated intravascular coagulation); neither was considered related to [^177^Lu]Lu-PNT2002. The fatal event occurred 54 days after administration of the first cycle and prior to the second, leading to premature discontinuation of treatment. Grade ≥3 TEAEs were reported for 25.9% (7/27) of participants and treatment related for two participants. These included anemia in both participants and thrombocytopenia and neutropenia in one participant. There were no treatment-related serious TEAEs, treatment-related events leading to treatment discontinuation, or treatment-related deaths. No safety signals were observed among the subset of participants who received a predicted renal dose >23 Gy (**Supplementary Table 2**).

**Table 4:** Overall incidence of treatment-emergent adverse events (TEAEs).

Event	All TEAEs, n (%)	Treatment-related TEAEs, n (%)
TEAEs	25 (92.6)	14 (51.9)
TEAEs of grade 3 or higher	7 (25.9)	2 (7.4)
Serious TEAEs	5 (18.5)	0 (0.0)
TEAEs leading to discontinuation of [^177^Lu]Lu-PNT2002	1 (3.7)	0 (0.0)
TEAEs leading to delay of [^177^Lu]Lu-PNT2002 administration*	2 (7.4)	1 (3.7)
TEAEs leading to death	1 (3.7)	0 (0.0)

*Reflects intended action at time of AE.

**Table 5:** Incidence of common (≥10%) TEAEs by preferred term and maximum grade.

Event	All TEAEs, n (%)			Treatment-related TEAEs, n (%)		
	Grade 1	Grade 2	Grade 3	Grade 1	Grade 2	Grade 3
Dry Mouth	7 (25.9)	0	0	6 (22.2)	0	0
Fatigue	4 (14.8)	3 (11.1)	0	3 (11.1)	2 (7.4)	0
Nausea	4 (14.8)	2 (7.4)	0	3 (11.1)	2 (7.4)	0
Anemia	1 (3.7)	1 (3.7)	4 (14.8)	1 (3.7)	1 (3.7)	2 (7.4)
Hematuria	2 (7.4)	0	3 (11.1)	0	0	0
Arthralgia	2 (7.4)	2 (7.4)	0	0	0	0
Back pain	2 (7.4)	2 (7.4)	0	0	1 (3.7)	0
Pain in extremity	2 (7.4)	2 (7.4)	0	0	0	0
Decreased appetite	3 (11.1)	0	0	0	0	0
Dyspepsia	3 (11.1)	0	0	2 (7.4)	0	0
Headache	2 (7.4)	1 (3.7)	0	0	1 (3.7)	0
Musculoskeletal chest pain	3 (11.1)	0	0	0	0	0

### 3.3 Efficacy

**Figure 2:**
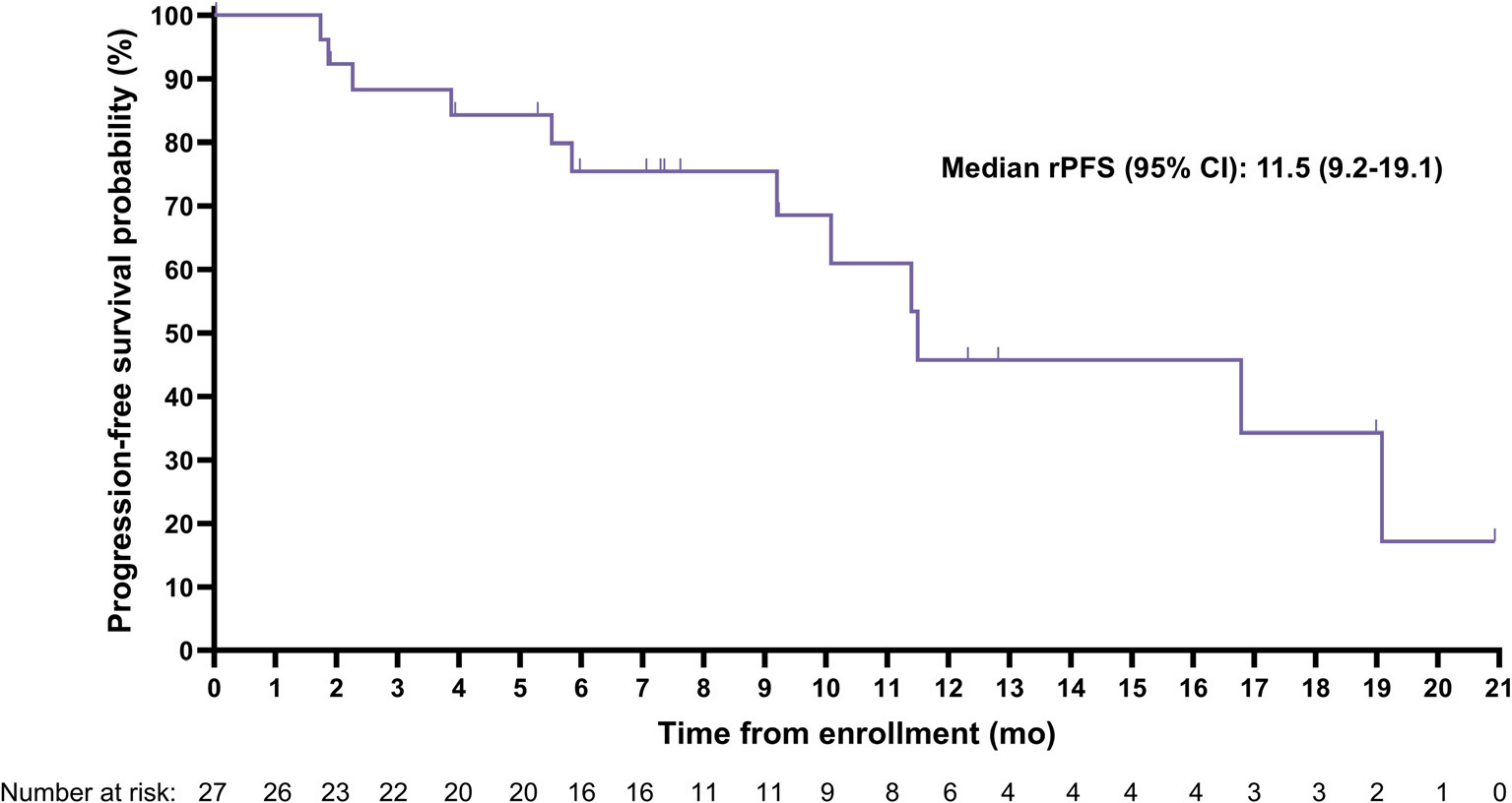
Kaplan-Meier curve for radiographic progression-free survival (rPFS) assessed by blinded independent central review.

**Figure 3:**
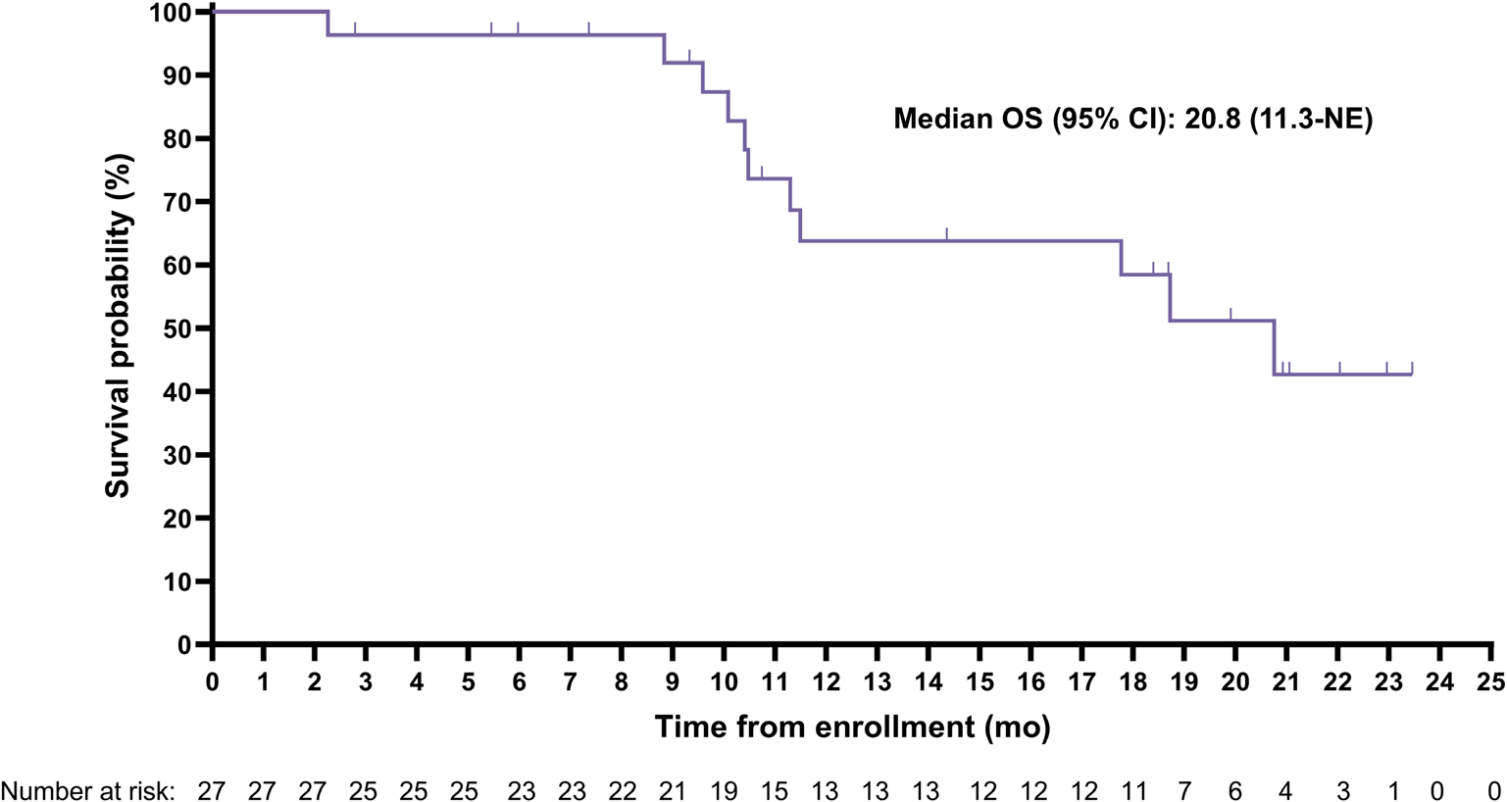
Kaplan-Meier curve for overall survival (OS).

The median (95% CI) rPFS was 11.5 (9.2-19.1) months with a median imaging follow-up time of 9.2 months (**Figure 2**). Median (95% CI) overall survival was 20.8 (11.3-Not evaluable) months with a median survival follow-up time of 19.9 months (**Figure 3**); 11 participants remained in long-term follow-up at the time of analysis.

**Figure 4:**
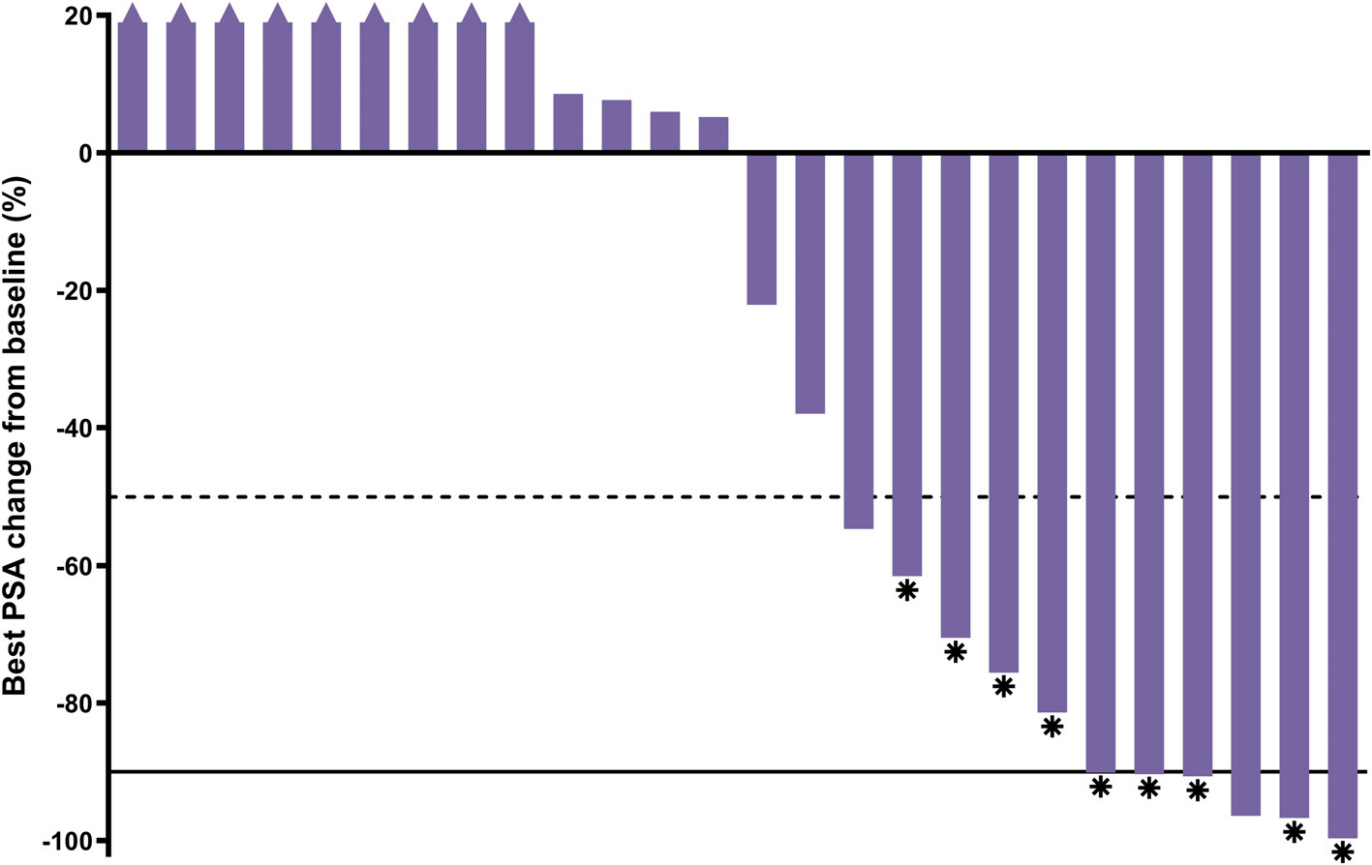
Best PSA percent change from baseline; n=26 (1 participant had no post-baseline data; only received 1 cycle). Asterisk = confirmed response; dashed line = 50% decline; solid line = 90% decline; triangle = increase >20% (truncated).

A PSA decline of ≥50% (PSA_50_) was achieved by 42.3% (11/26) of participants and confirmed per PCWG3 criteria for nine (34.6%) participants (**Figure 4**).

**Figure 5:**
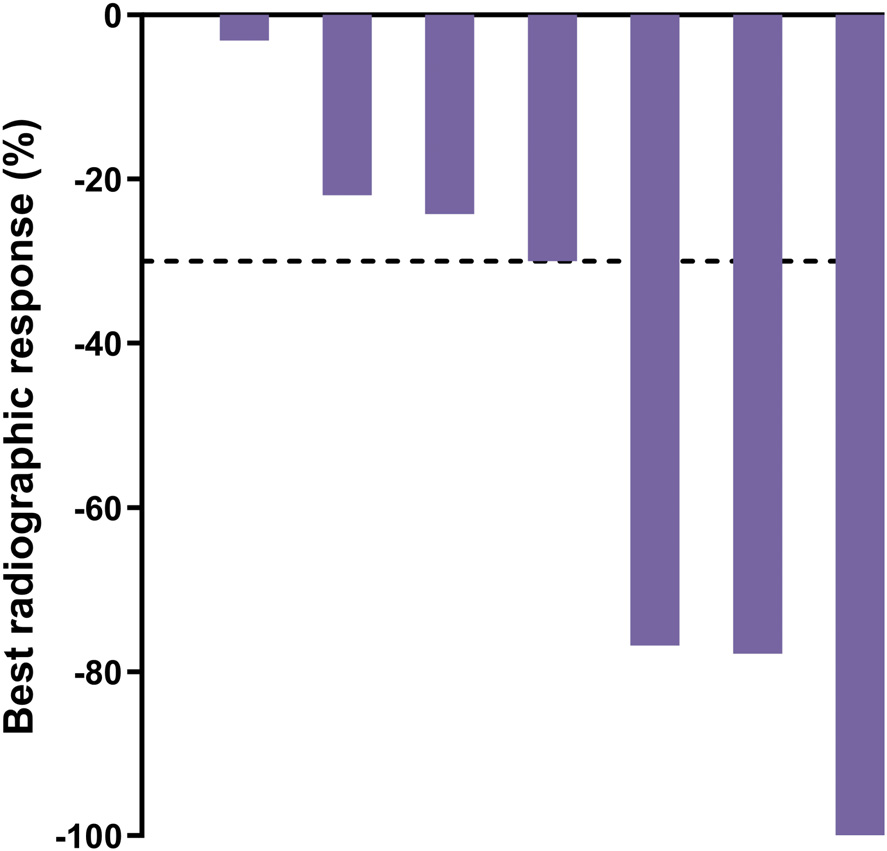
Best radiographic response by blinded independent central review for participants with measurable disease per RECIST v1.1 at baseline; n=7. Dashed line = 30% decline.

**Figure 6:**
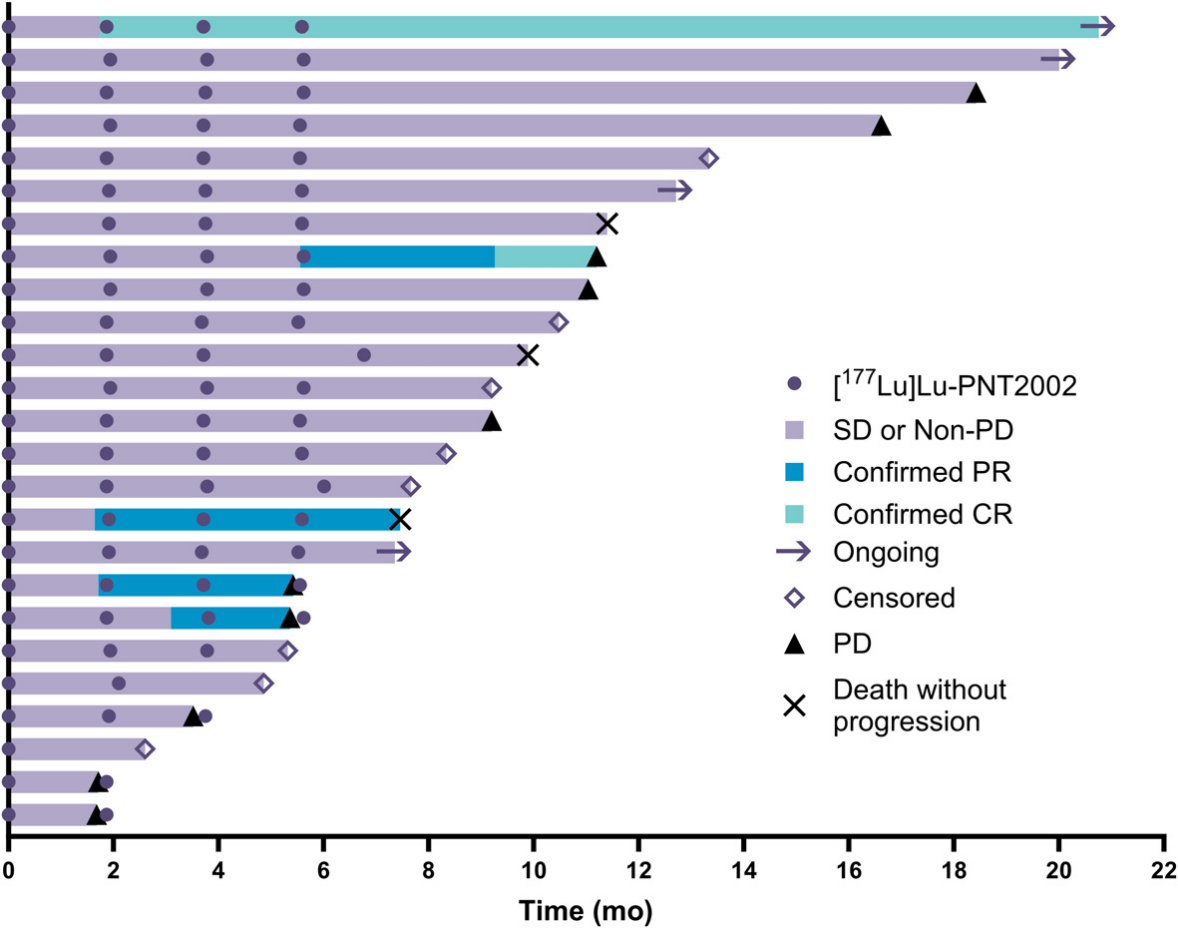
Swimmers’ plot of individual responses to treatment; n=25 (two participants did not have post-baseline imaging assessments). CR, complete response; PD, progressive disease; PR, partial response; SD, stable disease.

Ten participants had evaluable disease by RECIST v1.1 at baseline. Of these, two had confirmed complete responses, three had confirmed partial responses, three had stable disease, one had an unconfirmed partial response, and one had progressive disease, leading to a confirmed radiographic ORR by blinded independent central review of 50% (5/10). Baseline disease was measurable per RECIST v1.1 for seven participants, whose best radiographic responses are shown in **Figure 5**. Individual responses to treatment are illustrated for all 25 participants with at least one post-baseline radiographic assessment (**Figure 6**).

## 4 Discussion

The results from the lead-in portion of the SPLASH trial confirmed that [^177^Lu]Lu-PNT2002 at 6.8 GBq/cycle for 4 cycles had a favorable dosimetry and safety profile, as well as promising preliminary efficacy.

Tumor lesion specific absorbed dose, assessed for the subset of seven participants with adequate SPECT/CT imaging, was 2.1/4.3 (median/mean) Gy/GBq, which is similar to the 3.3 (median) and 3.2 (mean) Gy/GBq previously reported for [^177^Lu]Lu-PSMA-I&T (11, 13). However, in this study, cumulative absorbed dose was extrapolated from cycle 1 and is therefore likely slightly overestimated, as absorbed dose to tumors (though not normal organs) tends to decrease with each cycle for [^177^Lu]Lu-PSMA-I&T (14). Additionally, there is also some potential for overestimation in our normal organ dosimetry results as we used planar-only imaging for most participants and a single bed position for those with SPECT/CT. These techniques have the potential to overestimate activity due to overlap of gastrointestinal tract with kidneys and/or bone metastases with nearby organs at risk. The latter is particularly noteworthy here given the high frequency of bone metastases in mCRPC and the proximity of bone to key organs of interest, including kidneys and salivary and lacrimal glands.

Mean specific absorbed doses for red marrow calculated by different methods from two separate tissue types (red marrow contained in the lumbar spine, and blood contained in the heart contents) were low and remarkably similar (0.033 and 0.034 Gy/GBq), despite the limited number of participants with SPECT/CT available for the lumbar spine analysis and the unvalidated nature of the heart/blood planar-based analysis. However, both methods have limitations and dosimetry alone does not account for other critical predictors of hematological toxicity such as pretherapy bone marrow reserve or radiosensitivity (15).

Mean absorbed renal dose, calculated from a combination of planar-only and hybrid (planar + SPECT/CT) imaging, was 0.73 Gy/GBq, which is consistent with the 0.8 (median) and 0.72 Gy/GBq renal doses previously reported for [^177^Lu]Lu-PSMA-I&T (11, 13). The cumulative administered activity of 4 cycles at 6.8 GBq/cycle (27.2 GBq) extrapolated from cycle 1 yields an estimated mean cumulative absorbed renal dose of 19.9 Gy, which does not exceed the 23 Gy external beam radiation absorbed dose threshold set by the Food and Drug Administration to ensure <5% renal damage at 5 years (16). At the individual level, six participants had predicted cumulative renal doses exceeding the 23 Gy threshold; however, no additional safety signals were observed in this group. The 23 Gy renal threshold is based on external beam radiation data and is probably not appropriate for molecularly targeted radioligand therapy due to several important differences between these two types of radiation, including dose rate and spatial uniformity of dose deposition (17). However, there is evidence suggesting potential renal impairment during radioligand therapy with [^177^Lu]Lu-PSMA-I&T. Steinhelfer et al. reported on the long-term nephrotoxicity of [^177^Lu]Lu-radioligand therapy, while Schäfer et al. described cases of radiation nephropathy with a renal thrombotic microangiopathy-like picture following extensive [^177^Lu]Lu-PSMA therapy (18, 19). Bodei et al. have suggested that a 40 Gy biologically effective dose to kidney from [^177^Lu]Lu-radioligand therapy represents a more reliable threshold of toxicity in those without pre-existing renal conditions (20).

[^177^Lu]Lu-PNT2002 was very well tolerated, with the low incidence of significant myelosuppression observed for this lead-in cohort being particularly noteworthy. Overall, the safety profile was consistent with that previously reported for [^177^Lu]Lu-PSMA-I&T. No new or unexpected safety signals were observed.

Compelling anti-tumor activity was demonstrated with a PSA_50_ of 42.3% and a confirmed radiographic ORR of 50% among those with evaluable disease at baseline. Notably, the median rPFS of 11.5 months was considerably longer than the 3.5 to 4.2-month rPFS expected based on historical data for a second ARPI from trials conducted in similar populations (21, 22). Additionally, the median OS of 20.8 months observed for the SPLASH lead-in cohort reflects a more than 2-fold increase over the published median OS of 8.6 months for a second ARPI in chemo-naïve mCRPC patients (n=47) from a retrospective, multi-center cohort study (23). Interestingly, the median rPFS and OS were remarkably similar to those recently reported for [^177^Lu]Lu-PSMA-617 in a similar trial (24), despite the lower administered activity (6.8 vs 7.2 GBq/cycle) and fewer cycles (4 vs 6) used in SPLASH. The rationale behind this dose regimen is to reduce the overall treatment intensity by administering fewer cycles over a longer period compared to [^177^Lu]Lu-PSMA-617 in PSMAfore (every 6 weeks for 6 cycles) and VISION (every 6 weeks for 4 to 6 cycles) trials (24, 25). The prescribed activity administered from a complete regimen of [^177^Lu]Lu-PNT2002 resulted in lower estimated absorbed doses to several key organs, including the lacrimal glands (32.6 vs 92 Gy), salivary glands (9.2 vs 28 Gy), and red marrow (0.9 vs 1.5 Gy), relative to results from the VISION dosimetry substudy (26), and may ultimately minimize associated toxicities, although not in the kidney (19.9 vs 19 Gy) due to inherent differences in ligands (27). However, a lower dose and longer interval also has the potential to result in slightly lower response rates, though this cannot be conclusively determined in the absence of a trial directly comparing the dose regimes.

Limitations of the lead-in portion of SPLASH include the relatively small number of participants and single-arm design; both limit interpretability of rPFS and OS results. However, the design of this portion of SPLASH was suitable for its intended purpose of evaluating the treatment regimen by assessing dosimetry and clinical toxicity, as well as determining whether the planned number of cycles and activity per cycle were high enough to elicit the desired anti-tumor effect and low enough to avoid clinical toxicities. Now that the lead-in portion of the study has successfully confirmed the appropriateness of the regime, SPLASH has advanced to the randomized portion of the trial. More than 390 participants have been randomized 2:1 to receive [^177^Lu]Lu-PNT2002 or a second ARPI (with the option to cross over to [^177^Lu]Lu-PNT2002 after progression), providing both a contemporaneous comparator arm and sufficient power to determine the safety and efficacy of [^177^Lu]Lu-PNT2002.

## 5 Conclusion

In the 27-participant lead-in portion of the SPLASH trial, [^177^Lu]Lu-PNT2002 demonstrated a favorable dosimetry and safety profile, along with promising anti-tumor activity and efficacy. Accordingly, the efficacy and safety of [^177^Lu]Lu-PNT2002 at 6.8 GBq for 4 cycles in patients with PSMA-positive mCRPC who have progressed on an ARPI are being investigated in the multicenter, prospective, randomized portion of the SPLASH trial.

## Data Availability

The original contributions presented in the study are included in the article/**Supplementary Material**. Further inquiries can be directed to the corresponding author.
