# Supplementary material for: Initial clinical experience with [177Lu]Lu-PNT2002 radioligand therapy in metastatic castration-resistant prostate cancer: dosimetry, safety, and efficacy from the lead-in cohort of the SPLASH trial
Source: Front Oncol. 2025 Jan 7;14:1483953. doi: 10.3389/fonc.2024.1483953 (PMC11745944; doi:10.3389/fonc.2024.1483953)
Supplement: Supplementary file 1 [file DataSheet1.docx]

***Supplementary Material***

1. **Expanded Image Acquisition & Dosimetry Methods**

**1.1 PET/CT Camera Systems & Reconstruction Methods**

PET/CT camera systems used to determine PSMA positivity for study eligibility included the Discovery STE (GE Healthcare); Vereos Digital and Gemini TF PET/CT (Philips Medical Systems); and the Biograph Vision, Biograph mCT, Biograph 16, and Biograph 40 (Siemens). Scanner validation and qualification data were collected to ensure normalization of calibrations and accuracy of scanner standardized uptake value (SUV) measurements. Time of flight (if available) and iterative reconstructions were performed and corrections were applied for artifacts including attenuation correction, scatter correction, and corrections for random coincidences as needed.

**1.2 Image Acquisition & Region of Interest (ROI) Construction: Planar Images**

For normal organ dosimetry, whole-body planar images were collected for all participants at five time points following the first treatment: 0.5-2 h (pre-void), 24 h (±4 h), 48 h (±4 h), 72 h (±4 h), and 140-196 h. To calibrate the gamma camera and standardize the images, an imaging calibration standard containing approximately 37 MBq in 1 mL was placed near the patient’s feet for each image. Three energy windows were collected: a 20% photopeak window centered at 208 keV, a 10% lower scatter window contiguous with the lower end of the photopeak window centered at 178.3 keV, and a 10% upper scatter window contiguous with the upper end of the photopeak window centered at 240.8 keV. Regions of interest (ROI) were constructed manually. An example image of ROIs manually drawn on a planar image for a patient treated with PSMA-targeted radioligand therapy is displayed as Figure 2 in a publication by Yilmaz and colleagues (1). Planar count statistics for each ROI were determined for the photopeak and both scatter energy windows. Scatter correction for ROI counts was applied using a triple energy window technique as described in Medical Internal Radiation Dose (MIRD) Pamphlet 16 (2) using Equation 1, with scatter windows normalized to equal one-half the width of the primary window utilizing Equations 2 and 3.


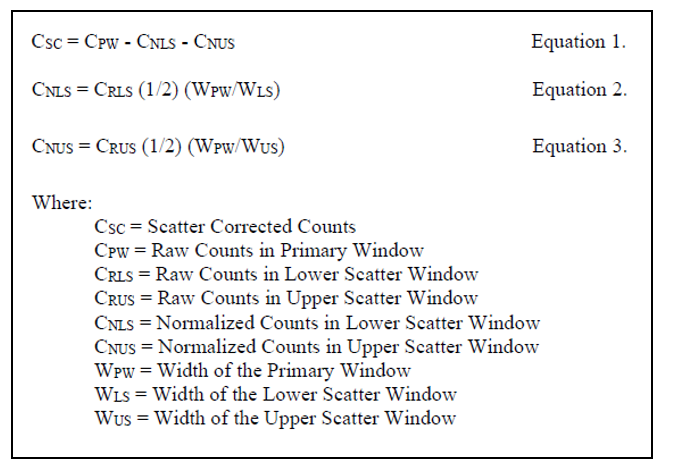


**1.3 Image Acquisition & ROI/Volume of Interest (VOI) Construction: SPECT/CT Images**

A single abdominal SPECT/CT image was collected at 48 ± 4 hours post injection. Sites performed appropriate corrections (attenuation, scatter, uniformity, etc.) and reconstruction methods to produce semi-quantitative SPECT/CT images (3). To determine calibration factors for normal organ dosimetry, a torso-sized (anterior to posterior depth similar to that of a human torso) phantom (without inserts) containing a known amount of activity in water was imaged for each camera system. ROIs/VOIs were manually constructed on the SPECT/CT images to determine the VOI count statistics in the kidneys and lumbar spine. ROIs/VOIs were constructed on the CT images and the resultant areas and thicknesses were utilized to determine patient-specific organ volumes for the kidneys.

For tumor dosimetry, a board-certified nuclear medicine physician selected up to 5 tumor lesions per participant. Selection was based on intensity, size, and image reproducibility and was limited to the field of view of the SPECT/CT images. If more than one organ system was affected, representative lesions were selected for each. Both bone and soft tissue metastases were included. Tumor ROIs/VOIs were manually constructed on the SPECT/CT images to determine the VOI count statistics. To compensate for partial volume effects, a second type of phantom was imaged. The second phantom was a torso-sized NEMA phantom with tumor-sized fillable inserts. The activity concentration ratio was approximately 1:10 between the tumor-sized fillable inserts and the background (i.e., the liquid surrounding the inserts) and the exact activity of each insert and of the background were determined. An imaging calibration standard (a known amount of activity sealed within a thin-walled container such as a small syringe or vial) was imaged alongside the phantom. The phantom SPECT/CT images were corrected and reconstructed by the site using exactly the same protocol as the patient SPECT/CT images. ROIs/VOI of the entire phantom were manually constructed, and the SPECT/CT image calibration factor was determined by taking the ratio of the resultant total counts per second to the total phantom activity at the time of the image (3). The calibration factor determined using the calibration source was checked for reasonable correspondence to the calibration factor determined using the total phantom to ensure that the SPECT image acquisition, correction, and reconstruction methods were reasonable. Recovery coefficients were determined by taking the ratio of the activity determined using VOIs constructed on each fillable insert to the assay determination of the activity actually contained in each insert.

**1.4 Activity Quantification: Planar Images**

Organ/tissue and whole-body activities were determined using the methodology as presented in the MIRD 16 document "Techniques for Quantitative Radiopharmaceutical Biodistribution Data Acquisition and Analysis for Use in Human Radiation Dose Estimates" (2), according to Equations 4-8. Equation 7 was replaced with direct decay correction and scan speed adjustment, in cases of a missing or problematic image reference standard.


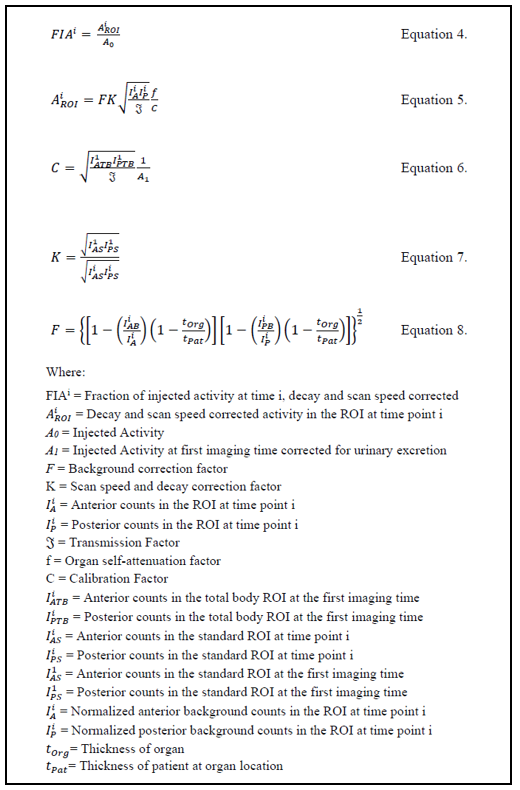


Tumor, organ, and body thicknesses for background subtraction were estimated using CT measurements or estimated from scaled model dimensions (4-5). Transmission factors for tumors, kidneys, and lumbar spine were determined using Equation 9, CT measurements and/or scaled model dimensions of the body thickness at the tumor, kidney, or lumbar spine locations, and the broad-beam attenuation coefficient of the isotope (6). Transmission factors for the whole body were also estimated using Equation 9, patient cross-sectional area determined from the first planar whole-body scan, the total body density found using reference man tissue masses and densities, and the broad-beam attenuation coefficient of the isotope (6). For organs/tissues other than tumors, kidneys, and lumbar spine, attenuation and background corrections were not performed (𝔍 and F assumed to be unity).


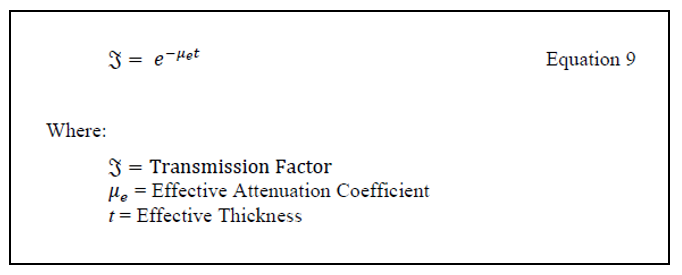


**1.5 Activity Quantification: SPECT/CT Images**

For the subset of participants with SPECT/CT imaging, activity in the tumors, kidneys, and lumbar spine were determined at the SPECT/CT imaging time using Equation 10. The result of this quantification was used to scale the tumor, kidney, and lumbar time-activity curves determined from the planar imaging according to methodology previously described (7-8, 3).


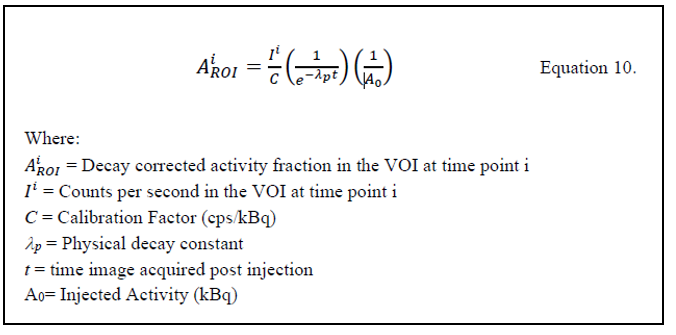


To compensate for partial volume effects, camera-specific recovery coefficients were determined from NEMA phantoms with insert activity concentration ratio to background of approximately 10:1. Tumor volume-specific recovery coefficients were derived by fitting the recovery coefficient – volume curves using non-linear regression with sums of exponentials of the form shown in subsequently in Equation 15. Corrections to ROI volumes for partial volume effects were performed using Equation 11.


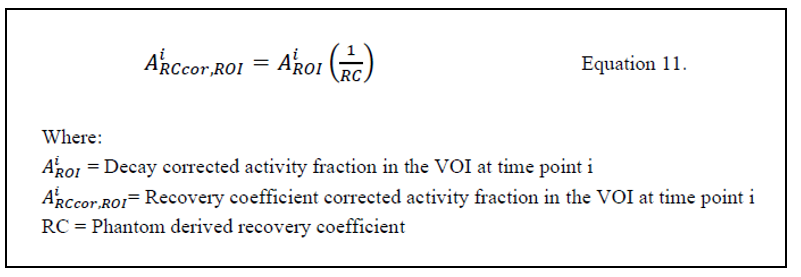


**1.6 Red Marrow Activity: Planar Images**

Since non-disease specific marrow uptake was not observed in the planar images in areas associated with marrow, a blood-based method to determine red marrow activity was applied as per the publication “EANM Dosimetry Committee Guidelines for Bone Marrow and Whole-Body Dosimetry” (9), and (10). Activity in the blood was estimated using a heart ROI as previously described (11), wherein the heart ROI activity is the background-subtracted sum of the total counts per second in the right and left atria and ventricles and the blood fraction of administered activity is calculated by dividing by the volume of the MIRD reference phantom heart (560 ml) scaled by the ratio of patient-specific total blood volume divided by a reference adult total blood volume of 5200 ml (11). A patient-specific blood volume was determined based on the work of Nadler (12) as shown in Equation 12, and a patient-specific red marrow volume was determined based on the ratio of red marrow mass to blood mass as described in ICRP 89 (13). Red marrow activity was estimated using the EANM recommended method (9-10) according to Equation 13, where a conservative value of the RMBLR (1.0) was assumed (10,14).


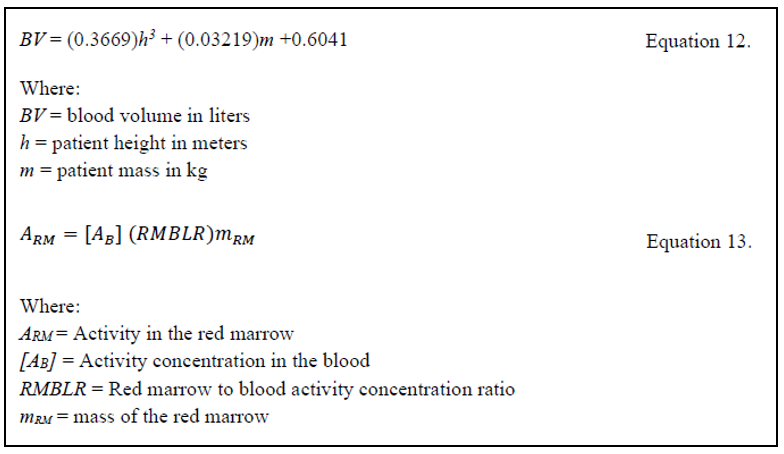


**1.7 Red Marrow Activity: SPECT/CT Images**

For the subset of participants with SPECT/CT imaging, red marrow activity was also determined based on a lumbar spine VOI. Activity in the lumbar spine was determined as described in the “Activity Quantification: SPECT/CT Images” section and scaled to represent total body red marrow by assuming that the lumbar spine contained 12.3% of the total marrow (13). A patient-specific red marrow mass was determined based on the ratio of the patient mass to the radiation transport phantom mass using Equation 14 as described in EANM Dosimetry Committee guidelines for bone marrow and whole-body dosimetry (9).


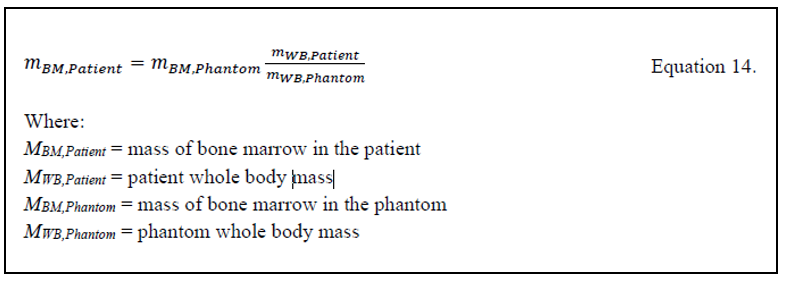


**1.8 Excretory Clearance & Remainder Activity**

Urinary excretion after injection and prior to the first imaging time was avoided unless necessary for the participant. If urine was voided, the site collected the entire void, measured the total volume, and an aliquot was assayed such that the total activity excreted in the urine prior to the first image could be determined by multiplication of the assay result in activity concentration by the total void volume. This activity was then subtracted from the injected activity, for use in the determination of the calibration factor using Equation 6.

**1.9 Source Organ/Tissue Time-Activity Biokinetic Modeling**

Organ and tissue activity data were decay corrected and fit in a least squares sense using non-linear regression with sums of exponentials of the form shown in Equation 15. Tumor data from the first timepoint were omitted from modeling due to low uptake. Organ activity counts were fit to exponential models considering the physical decay constant of the isotope, then time-integrated activity coefficients were determined using Equations 16 and 17.


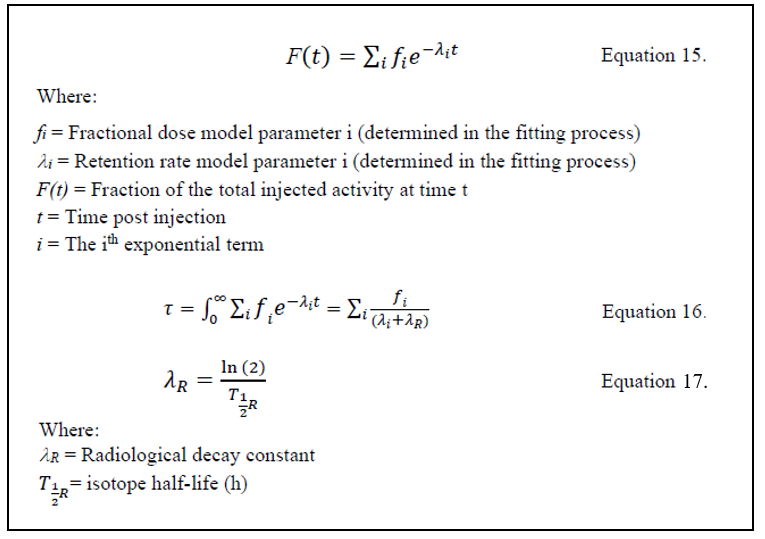


**1.10 Organ/Tissue Dosimetry Estimates**

The FDA cleared software OLINDA/EXM (15-16) v2.2 was used to estimate the radiation absorbed doses. OLINDA/EXM derives the emission information for the selected nuclide from a tabulated database (17). It then extracts absorbed fractions for all relevant target/source combinations for the selected radiation transport model from the tabulated phantom data (18). The emissions and the absorbed fractions are then used by the code to determine absorbed dose per unit activity (S-values) for all relevant target/source combinations as per the RADAR/MIRD methods for internal dosimetry (15-16, 19). OLINDA/EXM then utilizes these S-values and the time-integrated activity coefficients to produce final organ and tissue dose estimates. The OLINDA/EXM software has a complete series of dosimetry phantoms corresponding to different age “reference" humans (20). The same sex phantom closest in mass to the patient was selected.

Lacrimal gland dosimetry is not included in OLINDA. Lacrimal gland dosimetry was determined using the standard MIRD/RADAR methodology. Each lacrimal gland was assumed to be a 0.7 g sphere based on anatomical information from 11 references (21-31). S-value for the sphere was determined using non-linear regression of sphere S-values for the isotope obtained from the OLINDA sphere model. The lacrimal glands time course of activity and residence times were determined by the same methodology described for other organs.

For the subset of participants with SPECT/CT imaging, estimated kidney absorbed doses were adjusted for patient-specific kidney mass based on CT measurements of kidney volume and tumor dosimetry was determined using the standard MIRD/RADAR methodology. S-values for the tumors were determined using CT derived tumor volumes, and tumor tissue type as input to the IDAC dosimetry code (32).

**References**

1. Yilmaz B, Nisli S, Ergul N, Gursu RU, Acikgoz O, Çermik TF. Effect of external cooling on ^177^Lu-PSMA uptake by the parotid glands. *J Nucl Med*. (2019) 60:1388-93. doi: 10.2967/jnumed.119.226449
2. Siegel JA, Thomas SR, Stubbs JB, Stabin MG, Hays MT, Koral KF, et al. MIRD pamphlet no. 16: Techniques for quantitative radiopharmaceutical biodistribution data acquisition and analysis for use in human radiation dose estimates. *J Nucl Med*. (1999) 40:37S-61S.
3. Ljungberg M, Celler A, Konijnenberg MW, Eckerman KF, Dewaraja YK, Sjögreen-Gleisner K, et al. MIRD pamphlet no. 26: Joint EANM/MIRD guidelines for quantitative 177Lu SPECT applied for dosimetry of radiopharmaceutical therapy. *J Nucl Med*. (2016) 57:151-62. doi: 10.2967/jnumed.115.159012
4. Cristy, M. and Eckerman, K. (1987). Specific absorbed fractions of energy at various ages from internal photons sources. ORNL/TM-8381 V1-V7. Oak Ridge National Laboratory, Oak Ridge, TN.
5. Ogawa K. [Method of determining body thickness from weight and height]. *Nihon Hoshasen Gijutsu Gakkai Zasshi*. (2009) 65:50-6. doi: 10.6009/jjrt.65.50
6. Bailey DL, Hennessy TM, Willowson KP, Henry EC, Chan DL, Aslani A, et al. In vivo quantification of (177)Lu with planar whole-body and SPECT/CT gamma camera imaging. *EJNMMI Phys*. (2015) 2:20. doi: 10.1186/s40658-015-0123-2
7. Belli ML, Mezzenga E, Di Iorio V, Celli M, Caroli P, Canali E, et al. A whole body dosimetry protocol for peptide-receptor radionuclide therapy (PRRT): 2D planar image and hybrid 2D+3D SPECT/CT image methods. *J Vis Exp*. (2020) Apr 24:(158). doi: 10.3791/60477
8. Dewaraja YK, Frey EC, Sgouros G, Brill AB, Roberson P, Zanzonico PB, et al. MIRD pamphlet No. 23: quantitative SPECT for patient-specific 3-dimensional dosimetry in internal radionuclide therapy. *J Nucl Med*. (2012) 53:1310-25. doi: 10.2967/jnumed.111.100123
9. Hindorf C, Glatting G, Chiesa C, Lindén O, Flux G; EANM Dosimetry Committee. EANM Dosimetry Committee guidelines for bone marrow and whole-body dosimetry. *Eur J Nucl Med Mol Imaging*. (2010) 37:1238-50. doi: 10.1007/s00259-010-1422-4
10. Siegel JA. Establishing a clinically meaningful predictive model of hematologic toxicity in nonmyeloablative targeted radiotherapy: practical aspects and limitations of red marrow dosimetry. *Cancer Biother Radiopharm*. (2005) 20:126-40. doi: 10.1089/cbr.2005.20.126. Erratum in: *Cancer Biother Radiopharm*. (2005) 20(3):371.
11. Erwin W. A novel heart-contents image-based Yttrium-90-Zevalin red marrow residence time estimator. *J Nucl Med*. (2010). 51 (Suppl 2): 1323.
12. Nadler SB, Hidalgo JH, Bloch T. Prediction of blood volume in normal human adults. *Surgery*. (1962) 51:224-32.
13. Basic anatomical and physiological data for use in radiological protection: reference values. A report of age- and gender-related differences in the anatomical and physiological characteristics of reference individuals. ICRP Publication 89. *Ann ICRP*. (2002) 32:5-265.
14. Gosewisch A, Delker A, Tattenberg S, Ilhan H, Todica A, Brosch J, et al. Patient-specific image-based bone marrow dosimetry in Lu-177-[DOTA^0^,Tyr^3^]-Octreotate and Lu-177-DKFZ-PSMA-617 therapy: investigation of a new hybrid image approach. *EJNMMI Res*. (2018) 8:76. doi: 10.1186/s13550-018-0427-z
15. Stabin MG, Sparks RB, Crowe E. OLINDA/EXM: the second-generation personal computer software for internal dose assessment in nuclear medicine. *J Nucl Med*. (2005) 46:1023-7.
16. Stabin MG and Farmer A. OLINDA/EXM 2.0: The new generation dosimetry modeling code. J Nucl Med. (2012) 53 (Suppl 1):585.
17. Stabin MG, da Luz LC. Decay data for internal and external dose assessment. *Health Phys*. (2002) 83:471-5. doi: 10.1097/00004032-200210000-00004
18. Stabin MG, Emmons-Keenan MA, Segars WP, Fernald MJ. The Vanderbilt University Reference Adult and Pediatric Phantom Series. In: Xu XG and Eckerman KF, editors. Reference Adult and Pediatric Phantom Series: Handbook of Anatomical Models for Radiation Dosimetry. Boca Raton, FL: CRC Press (2009). p. 337–346.
19. Loevinger R, Budinger TF, Watson EE eds. MIRD Primer For Absorbed Dose Calculations. New York: Society of Nuclear Medicine (1991).
20. Stabin MG, Siegel JA. Physical models and dose factors for use in internal dose assessment. *Health Phys*. (2003) 85:294-310. doi: 10.1097/00004032-200309000-00006
21. Bingham CM, Castro A, Realini T, Nguyen J, Hogg JP, Sivak-Callcott JA. Calculated CT volumes of lacrimal glands in normal Caucasian orbits. *Ophthalmic Plast Reconstr Surg*. (2013) 29:157-159.
22. Bingham CM, Harris MA, Realini T, Nguyen J, Hogg JP, Sivak-Callcott JA. Calculated computed tomography volumes of lacrimal glands and comparison to clinical findings in patients with thyroid eye disease. *Ophthalmic Plast Reconstr Surg*. (2014) 30:116-8. doi: 10.1097/IOP.0000000000000015
23. Bulbul E, Yazici A, Yanik B, Yazici H, Demirpolat G. Evaluation of lacrimal gland dimensions and volume in Turkish population with computed tomography. *J Clin Diagn Res*. (2016) 10:TC06-8. doi: 10.7860/JCDR/2016/16331.7207
24. Gao Y, Moonis G, Cunnane ME, Eisenberg RL. Lacrimal gland masses. *AJR Am J Roentgenol*. (2013) 201:W371-81. doi: 10.2214/AJR.12.9553
25. Hänscheid H. Analysis and evaluation of dosimetry data from ^177^Lu-PSMA-617-therapies. Klinik und Poliklinik für Nuklearmedizin. (2018).
26. Hohberg M, Eschner W, Schmidt M, Dietlein M, Kobe C, Fischer T, et al. Lacrimal glands may represent organs at risk for radionuclide therapy of prostate cancer with [(177)Lu]DKFZ-PSMA-617. *Mol Imaging Biol*. (2016) 18:437-45. doi: 10.1007/s11307-016-0942-0
27. Okamoto S, Thieme A, Allmann J, D'Alessandria C, Maurer T, Retz M, et al. Radiation dosimetry for ^177^Lu-PSMA I&T in metastatic castration-resistant prostate cancer: Absorbed dose in normal organs and tumor lesions. *J Nucl Med*. (2017) 58:445-50. doi: 10.2967/jnumed.116.178483
28. Soundy RG, Tyrrell DA, Pickett RD, Stabin M. The radiation dosimetry of 99Tcm-exametazime. *Nucl Med Commun*. (1990) 11:791-9. doi: 10.1097/00006231-199011000-00008
29. Tamboli DA, Harris MA, Hogg JP, Realini T, Sivak-Callcott JA. Computed tomography dimensions of the lacrimal gland in normal Caucasian orbits. *Ophthalmic Plast Reconstr Surg*. (2011) 27:453-6. doi: 10.1097/IOP.0b013e31821e9f5d
30. Totuk OMG, Kalkay AB, Kabadayi K, Demir MK, Barut C. Evaluation of lacrimal gland dimensions with mr imaging in a Turkish population sample. *Int J Morphol*. (2018) 36:1431-38.
31. Yazici A, Bulbul E, Yazici H, Sari E, Tiskaoglu N, Yanik B, et al. Lacrimal gland volume changes in unilateral primary acquired nasolacrimal obstruction. *Invest Ophthalmol Vis Sci*. (2015) 56:4425-9. doi: 10.1167/iovs.15-16873
32. Andersson M, Johansson L, Eckerman K, Mattsson S. IDAC-Dose 2.1, an internal dosimetry program for diagnostic nuclear medicine based on the ICRP adult reference voxel phantoms. *EJNMMI Res*. (2017) 7:88. doi: 10.1186/s13550-017-0339-3
