# Supplementary material for: Initial clinical experience with [177Lu]Lu-PNT2002 radioligand therapy in metastatic castration-resistant prostate cancer: dosimetry, safety, and efficacy from the lead-in cohort of the SPLASH trial
Source: Front Oncol. 2025 Jan 7;14:1483953. doi: 10.3389/fonc.2024.1483953 (PMC11745944; doi:10.3389/fonc.2024.1483953)
Supplement: Supplementary file 2 [file Table1.docx]

**Supplementary Table 1. Kidney Activity at Time of SPECT/CT Image**

| **Participant** | **Planar** | **SPECT/CT** | **Ratio** | **Percent Difference** |
| --- | --- | --- | --- | --- |
| 1 | 1.7% | 1.8% | 0.956 | -4.4% |
| 2 | 2.3% | 2.0% | 1.153 | 15.3% |
| 3 | 2.4% | 2.3% | 1.057 | 5.7% |
| 4 | 1.7% | 2.0% | 0.864 | -13.6% |
| 5 | 2.7% | 2.3% | 1.182 | 18.2% |
| 6 | 2.1% | 2.4% | 0.848 | -15.2% |
| 7* | 3.9% | 2.0% | 1.955 | 95.5% |

*Kidneys were highly obscured by overlap of gastrointestinal activity in the planar images leading to gastrointestinal activity counts contributing to the quantification of the kidney regions of interest.
