# Supplementary material for: Initial clinical experience with [177Lu]Lu-PNT2002 radioligand therapy in metastatic castration-resistant prostate cancer: dosimetry, safety, and efficacy from the lead-in cohort of the SPLASH trial
Source: Front Oncol. 2025 Jan 7;14:1483953. doi: 10.3389/fonc.2024.1483953 (PMC11745944; doi:10.3389/fonc.2024.1483953)
Supplement: Supplementary file 3 [file Table2.docx]

**Supplementary Table 2. Treatment-related Adverse Events for Participants Who Received a Predicted Renal Dose >23 Gy**

| **Participant** | **Predicted Renal Dose, Gy** | **Treatment-related Adverse Events, Preferred Term (grade/duration)** |
| --- | --- | --- |
| 1 | 40.8 | Anaemia (G2/90 days); thrombocytopenia (G1/79 days); anaemia (G2/2 days); anaemia (G2/20 days); anaemia (G3/2 days); anaemia (G2/103 days) |
| 2 | 27.2 | Dry mouth (G1/140 days); hypophosphataemia (G2/28 days) |
| 3 | 27.2 | Hyperphosphataemia (G1/13 days); acute kidney injury (G3/2 days)* |
| 4 | 25.3 | None reported |
| 5 | 24.2 | None reported |
| 6 | 23.4 | Headache (G2/4 days); nausea (G2/3 days); abdominal pain (G2/3 days) |

*This event was attributed mainly to poor fluid intake and diuretic treatment but was also deemed possibly related to [^177^Lu]Lu-PNT2002 treatment.
